# Personalized Risk Assessment for Prevention and Early Detection of Breast Cancer: Integration and Implementation (PERSPECTIVE I&I)

**DOI:** 10.3390/jpm11060511

**Published:** 2021-06-04

**Authors:** Jennifer D. Brooks, Hermann H. Nabi, Irene L. Andrulis, Antonis C. Antoniou, Jocelyne Chiquette, Philippe Després, Peter Devilee, Michel Dorval, Arnaud Droit, Douglas F. Easton, Andrea Eisen, Laurence Eloy, Samantha Fienberg, David Goldgar, Eric Hahnen, Yann Joly, Bartha Maria Knoppers, Aisha Lofters, Jean-Yves Masson, Nicole Mittmann, Jean-Sébastien Paquette, Nora Pashayan, Rita Schmutzler, Tracy Stockley, Sean V. Tavtigian, Meghan J. Walker, Michael Wolfson, Anna Maria Chiarelli, Jacques Simard

**Affiliations:** 1 Dalla Lana School of Public Health, University of Toronto, Toronto, ON M5S 1A1, Canada; jennifer.brooks@utoronto.ca (J.D.B.); meghan.walker@ontariohealth.ca (M.J.W.); 2 CHU de Québec-Université Laval Research Center, Québec City, QC G1V 4G2, Canada; hermann.nabi@crchudequebec.ulaval.ca (H.H.N.); jocelyne.chiquette@chudequebec.ca (J.C.); michel.dorval@crchudequebec.ulaval.ca (M.D.); jean-yves.masson@crchudequebec.ulaval.ca (J.-Y.M.); 3 Department of Social and Preventive Medicine, Faculty of Medicine, Université Laval, Québec City, QC G1V 0A6, Canada; 4 Université Laval Cancer Research Center, Québec City, QC G1R 3S3, Canada; 5 Department of Molecular Genetics, Lunenfeld-Tanenbaum Research Institute, Sinai Health System, University of Toronto, Toronto, ON M5G 1X5, Canada; andrulis@lunenfeld.ca; 6 Centre for Cancer Genetic Epidemiology, Department of Public Health and Primary Care, School of Clinical Medicine, University of Cambridge, Cambridge CB1 8RN, UK; aca20@medschl.cam.ac.uk (A.C.A.); dfe20@medschl.cam.ac.uk (D.F.E.); 7 Department of Family Medicine and Emergency Medicine, Faculty of Medicine, Université Laval, Québec City, QC G1V 4G2, Canada; jean-sebastien.paquette@fmed.ulaval.ca; 8 Department of Physics, Engineering Physics and Optics, Faculty of Science and Engineering, Université Laval, Québec City, QC G1V 0A6, Canada; philippe.despres@phy.ulaval.ca; 9 Department of Human Genetics, Leiden University Medical Center, 2333 ZA Leiden, The Netherlands; p.devilee@lumc.nl; 10 Faculty of Pharmacy, Université Laval, Québec City, QC G1V 4G2, Canada; 11 CISSS de Chaudière-Appalaches Research Center, Lévis, QC G6V 3Z1, Canada; 12 Department of Molecular Medicine, Faculty of Medicine, Université Laval, Québec City, QC G1V 4G2, Canada; arnaud.droit@crchudequebec.ulaval.ca; 13 Sunnybrook Health Science Center, Toronto, ON M4N 3M5, Canada; andrea.eisen@sunnybrook.ca (A.E.); nicole.mittmann@cadth.ca (N.M.); 14 Programme Québécois de Cancérologie, Ministère de la Santé et des Services Sociaux, Québec City, QC G1S 2M1, Canada; laurence@laurenceeloy.com; 15 Ontario Health, Cancer Care Ontario, Toronto, ON M5G 2L3, Canada; samantha.fienberg@grhosp.on.ca; 16 Department of Oncological Sciences and Huntsman Cancer Institute, University of Utah, Salt Lake City, UT 84112, USA; david.goldgar@hsc.utah.edu (D.G.); sean.tavtigian@hci.utah.edu (S.V.T.); 17 Centre for Familial Breast and Ovarian Cancer, Center for Integrated Oncology, Medical Faculty, University Hospital Cologne, 50937 Köln, Germany; eric.hahnen@uk-koeln.de (E.H.); rita.schmutzler@uk-koeln.de (R.S.); 18 Centre of Genomics and Policy, McGill University, Montreal, QC H3A 0G1, Canada; yann.joly@mcgill.ca (Y.J.); bartha.knoppers@mcgill.ca (B.M.K.); 19 Women’s College Research Institute, Toronto, ON M5G 1N8, Canada; aisha.lofters@utoronto.ca; 20 Canadian Agency for Drugs and Technologies in Health, Toronto, ON M5H 3Y9, Canada; 21 Department of Applied Health Research, Institute of Epidemiology and Healthcare, University College London, London WC1E 6BT, UK; n.pashayan@ucl.ac.uk; 22 Division of Clinical Laboratory Genetics, University Health Network, Toronto, ON M5G 2C4, Canada; tracy.stockley@uhn.ca; 23 Department of Laboratory Medicine and Pathobiology, University of Toronto, Toronto, ON M5S 1A8, Canada; 24 School of Epidemiology and Public Health, University of Ottawa, Ottawa, ON K1G 5Z3, Canada; michael.wolfson@uottawa.ca

**Keywords:** breast cancer, screening, risk prediction, risk stratification

## Abstract

Early detection of breast cancer through screening reduces breast cancer mortality. The benefits of screening must also be considered within the context of potential harms (e.g., false positives, overdiagnosis). Furthermore, while breast cancer risk is highly variable within the population, most screening programs use age to determine eligibility. A risk-based approach is expected to improve the benefit-harm ratio of breast cancer screening programs. The PERSPECTIVE I&I (Personalized Risk Assessment for Prevention and Early Detection of Breast Cancer: Integration and Implementation) project seeks to improve personalized risk assessment to allow for a cost-effective, population-based approach to risk-based screening and determine best practices for implementation in Canada. This commentary describes the four inter-related activities that comprise the PERSPECTIVE I&I project. 1: Identification and validation of novel moderate to high-risk susceptibility genes. 2: Improvement, validation, and adaptation of a risk prediction web-tool for the Canadian context. 3: Development and piloting of a socio-ethical framework to support implementation of risk-based breast cancer screening. 4: Economic analysis to optimize the implementation of risk-based screening. Risk-based screening and prevention is expected to benefit all women, empowering them to work with their healthcare provider to make informed decisions about screening and prevention.

## 1. Introduction

Breast cancer is a leading cause of morbidity and mortality in women worldwide [1,2], with significant care and cost assumed by the public health care sector [3,4,5]. In Canada, mortality attributable to breast cancer has fallen by ~48% since the peak in 1986, mainly due to improved treatments and earlier diagnosis resulting from mammography screening [1].

Organizations responsible for publishing breast cancer screening recommendations, including the Canadian Task Force on Preventive Health Care, agree that women between the ages of 50–74 years should receive routine mammography screening [6,7,8]. Breast cancer screening, as currently practiced, uses age as the primary criterion for eligibility and is primarily targeted at women over 50 years of age. There is still considerable debate on the optimal screening interval, and whether younger women would also benefit from screening mammograms. The reason for this controversy is the need to balance the benefits of regular screening with potential harms including false-positive and false-negative findings, overdiagnosis, overtreatment, and the associated anxiety and unnecessary costs [8,9,10].

Breast cancer risk is highly variable, such that a large proportion of breast cancer cases occur in a minority of women who are most susceptible [11,12]. Most Canadian breast screening programs target women between the ages of 50 and 74 years, with mammograms every two years. Beyond this, many programs also have recommendations for younger (<50 years) and older (>74 years) women, as well as annual mammography for women at higher risk (e.g., those with a family history of breast cancer or dense breasts) [13,14]. Some programs also recommend screening with mammography, MRI and/or ultrasound (starting prior to age 50) for women identified as high-risk (e.g., greater than 20–30% lifetime risk), and/or refer these individuals for genetic testing [6,13,15,16,17,18]. Women are primarily identified as high risk based on having a family history of the disease, followed by testing for *BRCA1* and *BRCA2* mutations. This approach has the limitation of missing women without a known family history but with a significant genetic predisposition. Additional risk genes beyond *BRCA1* and *BRCA2* have been identified; however, commercially available testing panels include genes with uncertain impacts on risk [19,20,21]. This leads to significant challenges in the genetic counselling and clinical management of women who carry these variants. Beyond variation in rare and moderate penetrance genes, the discovery of many common genetic variants associated with breast cancer risk has led to development of polygenic risk scores (PRS) that can predict risk [22,23].

Multiple breast cancer risk prediction models have been developed and validated in a variety of settings [24,25,26,27], and are continually being updated to incorporate advances in the genomics and epidemiology of the disease. Recent versions of some models, such as the Breast and Ovarian Analysis of Disease Incidence and Carrier Estimation Algorithm (BOADICEA), now include mammographic density, one of the strongest risk factors for breast cancer, in addition to pathogenic variants in multiple genes and PRS [26]. There is increasing interest in the application of these models for risk stratification to inform recommendations for screening and prevention. This approach could be applied to both women at high risk, seen in cancer genetic clinics, as well as women in the general population.

Implementation of a risk-based approach to screening may bring new socio-ethical and legal challenges for women, health professionals and decision makers [28]. Additional legal duties for health professionals managing risk assessment, as well as potential issues surrounding the compliance of risk assessment tools with federal and provincial regulations will require careful consideration and foresight. Such anticipatory work will lead to the development of policies that appropriately address outstanding issues and that are also acceptable to end-users. It will also require significant economic considerations, given ever-present concerns about rising health care costs.

In this commentary, we describe how the PERSPECTIVE I&I (Personalized Risk Assessment for Prevention and Early Detection of Breast Cancer: Integration and Implementation) project seeks to improve personalized risk assessment, to offer cost-effective risk-based screening and interventions, and identify best practices to implementation within the context of the universal health care coverage provided by Canada’s health care programs.

## 2. Overview of the PERSPECTIVE I&I Project

**Figure 1:**
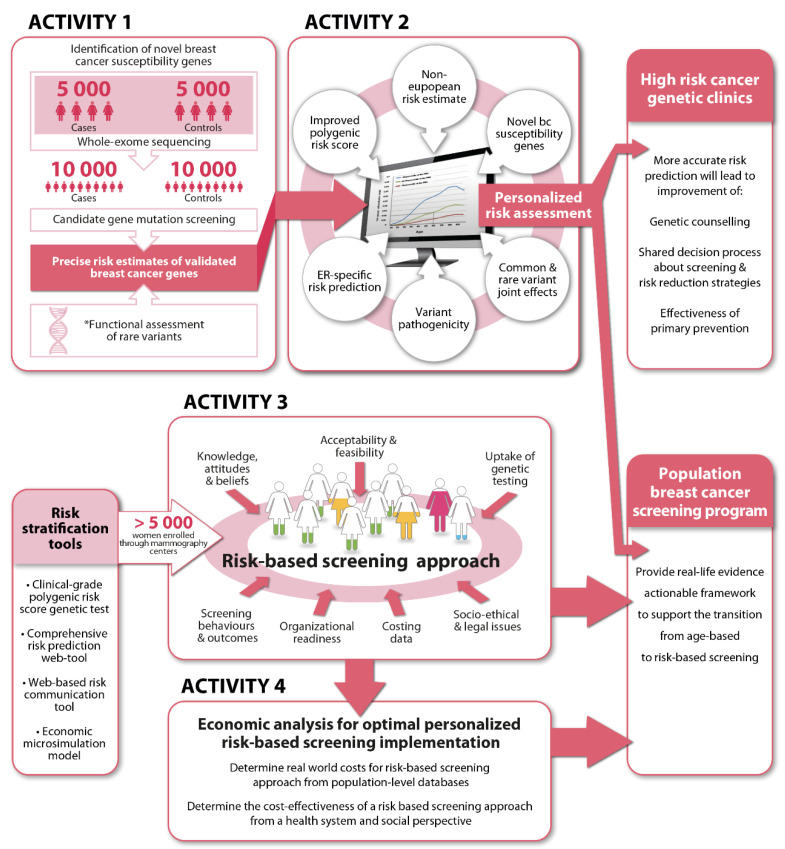
Overview of the PERSPECTIVE I&I project.

The PERSPECTIVE I&I project is comprised of four connected activities which are being undertaken largely in parallel. 1: Identification and validation of novel moderate- to high-risk breast cancer susceptibility genes through a well-powered whole exome sequencing (WES) case-control study, in order to develop a more comprehensive multi-gene panel test. 2: Improvement, validation and adaptation of a comprehensive risk prediction web-tool suitable to the Canadian context. 3: Development and piloting of a socio-ethical framework to support implementation of a personalized risk-based approach to breast cancer screening at the population level. 4: Economic analysis to optimize personalized risk-based screening implementation (Figure 1).

### 2.1. Identification and Validation of Novel Moderate- to High-Risk Breast Cancer Susceptibility Genes

Our understanding of the genetic susceptibility to breast cancer is essential to the assessment of a woman’s risk of the disease. Currently, variants in the known moderate and high-risk genes explain 15–20% of the heritability of breast cancer, while estimates for GWAS data suggest that common variants explain approximately 40% [19,20,21]. Only a small fraction of genes, mostly involved in DNA repair, have been investigated as potential susceptibility genes, raising the possibility that many other important susceptibility genes remain to be identified.

A major component of our PERSPECTIVE I&I project is focused on the identification of new breast cancer predisposition genes. This is being achieved through a large-scale whole exome sequencing (WES) study involving over 10,000 breast cancer cases and controls, where cases all have a family history of breast cancer and/or were diagnosed prior to age 50. The data resulting from the sequencing performed in PERSPECTIVE I&I will be combined with additional data generated through the EU-funded Horizon 2020 project BRIDGES (Breast Cancer Risk after Diagnostic Gene Sequencing, https://bridges-research.eu (accessed on 2 June 2021)). Thus, a combined analysis will be performed in a total of approximately 18,000 breast cancer cases and controls. This collaboration with the BRIDGES project almost doubles the size of the final sample set, which significantly increases the power to identify moderate-risk alleles. Candidate genes identified through this combined analysis will be subjected to validation, as part of PERSPECTIVE I&I, through targeted sequencing in a large sample set of 10,000 breast cancer cases and 10 000 controls from the Breast Cancer Association Consortium (http://bcac.ccge.medschl.cam.ac.uk (accessed on 2 June 2021)). The identification of new predisposition genes through this project will provide valuable information regarding the genetic susceptibility of breast cancer. This information can then be incorporated into clinical genetic tests used in the context of cancer genetic clinics to identify women at high risk of the disease. For genes that are confirmed, these data will also provide risk estimates that, upon further validation in larger studies, can then be incorporated in the updated risk models.

The sequencing efforts put forth to identify new breast cancer genes will be further supported by functional analysis of missense variants in selected breast cancer associated genes, focusing on those involved in DNA repair pathways [29,30]. Indeed, although the deleterious impact of truncating variants in known breast cancer susceptibility genes is well understood, the clinical significance of missense variants in such genes is often unknown and therefore represents an additional challenge for the clinical management of carriers of such variants of uncertain significance (VUS). An important component of our project involves the use of multiple functional assays of reported missense variants [30,31,32] as well as a high-throughput site-saturation mutagenesis approach to evaluate the functional impact of all possible missense substitutions in key breast cancer predisposition genes [33]. Collaboration and sharing of these results with the ENIGMA Consortium (Evidence-based Network for the Interpretation of Germline Mutant Alleles, https://enigmaconsortium.org (accessed on 2 June 2021)) will help improve the interpretation of missense VUS in these genes and accelerate their clinical translation.

#### Development of a Clinical Grade Genetic Test Including Validated Breast Cancer Susceptibility Genes and Polygenic Risk Score

In addition to the high and moderate risk breast cancer alleles, common low risk alleles also explain a significant proportion of the genetic susceptibility to breast cancer. While the risk conferred by individual common low risk SNPs (Single Nucleotide Polymorphisms) is not sufficiently large to be useful in risk prediction (e.g., RR < 1.3), their effects can be combined to provide a SNP profile or polygenic risk score (PRS), which can be incorporated into risk prediction models [34,35,36,37]. The application of a PRS to inform risk-stratified screening and other decisions (e.g., preventive interventions including prophylactic surgeries or chemoprevention) in the context of cancer genetics clinics requires the development of a clinical-grade genotyping assay suitable to generate a woman’s PRS. Particularly within the context of high-volume breast cancer screening programs, a test should be low-cost and high throughput, allowing for easy integration into other existing breast cancer genetic tests.

To develop a clinical grade PRS test in the PERSPECTIVE I&I project, we adapted the recently developed and validated 313 SNP PRS [23] to a next-generation sequencing (NGS) platform. NGS was chosen because of the ubiquity of its use in clinical laboratories, the potential for automation of technical procedures, the adaptability to genomic DNA from multiple sources (including saliva), and the ability to produce an inexpensive assay targeting the small genomic footprint of PRS SNP loci. NGS is also a versatile method that will allow for the addition of a SNP panel design into other existing NGS clinical assays, including those used for moderate- and high-penetrance genes (e.g., *BRCA1* and *BRCA2*). A major deliverable of the project will be the development of a new clinical-grade Breast Cancer Genetic Risk Multi-Gene test which will include the SNPs making up the PRS, as well as all coding exons of previously confirmed and newly identified breast cancer susceptibility genes. The new test, which will be intended for use in cancer genetic clinics, will address the need for a more comprehensive risk test to inform the genetic counselling of women at high risk of the disease.

### 2.2. Improvement, Validation and Adaptation of a Comprehensive Risk Prediction Web-Tool

Combining genetic and lifestyle/hormonal risk factors together can provide significant levels of breast cancer risk stratification [38,39]. The BOADICEA model [40] was originally developed to predict the risks of developing breast and ovarian cancer on the basis of *BRCA1* and *BRCA2* mutations and explicit cancer family history. It has subsequently been extended to incorporate the effects of rare pathogenic variants in the other established breast cancer susceptibility genes (*PALB2, CHEK2* and *ATM*), the effects of several lifestyle/hormonal and reproductive risk factors, mammographic density and the latest validated PRS for breast cancer [12,26,41]. This makes it the most comprehensive risk prediction model of its kind. Based on these risk factors, the model predicts substantial variation in absolute breast cancer risk among women in the general population, women with family history, and those with mutations in moderate- or high-risk genes [26]. This model has been validated for both predicting mutation carrier probabilities and future cancer risks [24,25,27,42].

The development and validation of BOADICEA, including the currently used PRS, has been based on data from women of European ancestry [23,26]. However, the distribution of risk factors can vary across populations, and both PRS distribution and effect size, have been shown to vary by ancestry [43]. As part of the CONFLUENCE project (https://dceg.cancer.gov/research/cancer-types/breast-cancer/confluence-project (accessed on 2 June 2021)), existing GWAS data and newly generated genome-wide genotyping data, from a total of 300,000 breast cancer cases and 300,000 controls of different races/ethnicities, will be used for the development of more powerful new subtype-specific PRS, to be incorporated into a later version of the model, in addition to improving the PRS in women of non-European ancestry. Furthermore, several additional breast cancer susceptibility genes have now been identified and will be incorporated into an extended version of BOADICEA. Novel genes identified through the WES performed in Activity 1 will also be incorporated. PERSPECTIVE I&I aims to customize and validate the extended version of BOADICEA to the diverse Canadian population. The validation will utilize two large prospective Canadian cohorts, the Canadian Partnership for Tomorrow’s Health study (CanPath; www.canpath.ca (accessed on 2 June 2021), formerly the Canadian Partnership for Tomorrow Project [CPTP]) [44] and the Canadian Longitudinal Study of Aging (CLSA, https://www.clsa-elcv.ca/ (accessed on 2 June 2021)). 

Finally, BOADICEA has been implemented in CanRisk (www.canrisk.org (accessed on 2 June 2021)), a web-tool that facilitates breast cancer risk calculations by health care professionals in the clinic [45]. This platform was developed following close consultations and usability assessments with key stakeholders at different levels of clinical care [46]. CanRisk has recently gained regulatory approval (CE marking) for use by healthcare professionals in the United Kingdom, and the European Economic Area (EEA) (released 6 January 2020) [45]. While the regulatory path for such web-tools in Canada is less clearly defined than in the UK/EEA regions [47,48,49,50,51], our team is at the forefront of the research and discussion to advance this issue and enable the use of CanRisk by Canadian health professionals [52].

### 2.3. Development of a Socio-Ethical Framework to Support Implementation of a Personalized Risk-Based Approach to Breast Cancer Screening

#### 2.3.1. Screening Cohorts

**Figure 2:**
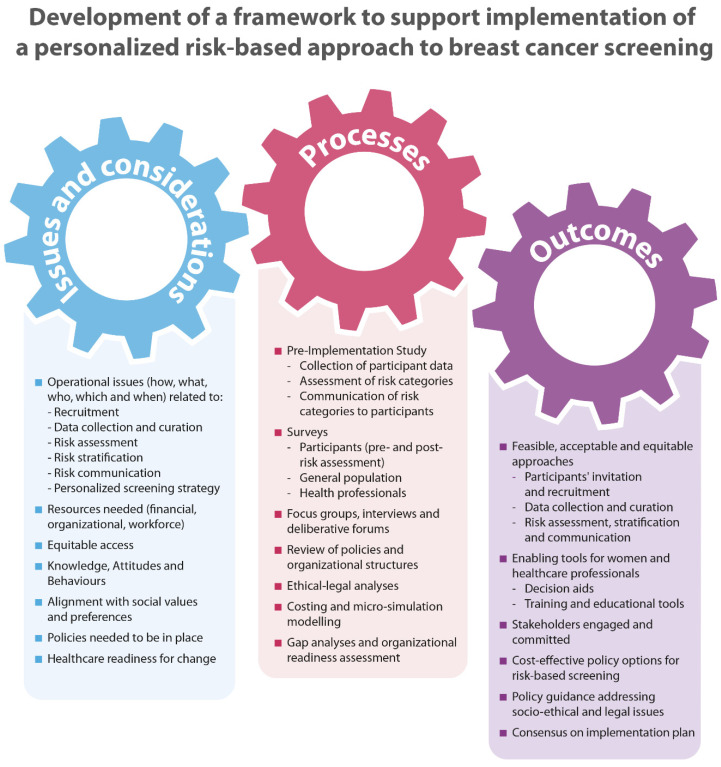
Framework to support implementation of a personalized risk-based approach to breast cancer screening.

To move beyond risk stratification within cancer genetic clinics and apply this approach at the population level, we need to assess acceptability, uptake, and effectiveness of a risk-based screening approach (Figure 2). To address these needs, we are conducting a pre-implementation study in >5000 women from Québec and Ontario, Canada. This study leverages the resources available through the existing screening programs including infrastructure, databases, links to primary care genetic clinics, and cancer registries [15,53,54].

**Figure 3:**
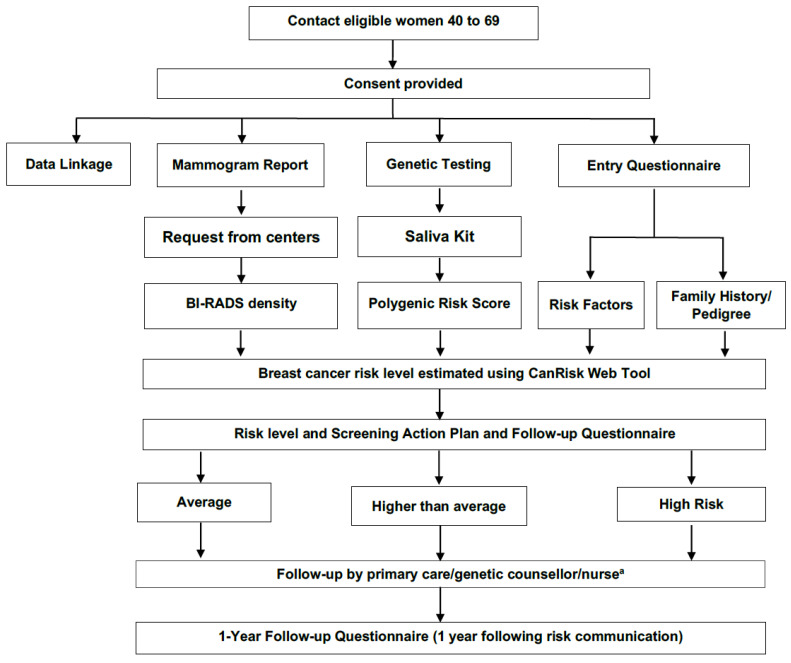
Data collection pathway for the PERSPECTIVE I&I Project. ^a^ upon request for Average and Higher than average risk categories.

Figure 3 provides an overview of data collection and follow-up, also described below. Broadly, unaffected women between the ages of 40–69 years are being solicited for recruitment through screening centers, family physicians and nurses, mailed invitations, social media (e.g., Facebook, Twitter), and study websites (https://etudeperspective.ca/; www.cancercareontario.ca/perspective (accessed on 2 June 2021)). In Québec, an online platform has been developed so that participants can complete the entire study process in a secure on-line environment. In Ontario, participants have the option of either paper or on-line questionnaires, allowing for the assessment of different recruitment and data collection strategies.

Eligible participants complete an entry questionnaire and provide consent for a DNA saliva test (to generate their PRS), and for release of screening mammogram reports (for mammographic density). The questionnaire captures the information needed for risk assessment using the CanRisk web tool, including detailed information on family history of cancers (first- and second-degree), anthropometric, hormonal, and lifestyle risk factors. The Predisposing, Reinforcing, and Enabling Constructs, in Educational Diagnosis and Evaluation (PRECEDE) model [55] was used to develop additional aspects of the questionnaire, which collects information related to a woman’s perceived risk of breast cancer, acceptability of genetic testing, and risk-based screening.

Following completion of the entry questionnaire, saliva kits for DNA sample collection are sent and returned by mail to a central clinical laboratory (University Health Network, Toronto, ON, Canada) for assessment of the PRS. Mammographic density, as reported by the radiologist according to the Breast Imaging, Reporting and Data System (BI-RADS) classification, is abstracted from recent mammogram reports. Questionnaire data, PRS, and mammographic density are then used to estimate 10-year breast cancer risks, using the CanRisk prediction tool [45].

All consenting women receive a personalized letter (via mail or secured website) which reports their risk category based on their 10-year age-dependent risk estimate. These three categories, referred to as “average”, “higher than average” and “high”, are based on a remaining lifetime risk (from age 30 to 80 years) of <15%, 15–24% and ≥25% respectively. The letter also provides details of a proposed screening action plan based on the risk category, age at risk assessment, and provincial breast screening guidelines (Table 1). Specifically, it is suggested that women estimated to be at average risk category aged 40–49 years, not undergo screening with mammography, while those who are age 50–69 years be screened every two years with a mammogram. Women estimated to be at higher-than-average risk category aged 40–49 years are encouraged to discuss screening options with their primary care provider, while it is proposed that those age 50–69 years undergo annual screening with mammogram. Finally, it is proposed that all women estimated to be in the high risk category (ages 40–69 years) undergo annual screening with both mammogram and MRI.

In Québec, the risk letter is also sent to the woman’s family physician, while in Ontario women can communicate this information to their primary care provider based on their preferences. In both provinces, women at high risk are contacted by a healthcare professional (e.g., family doctor, nurse, genetic counsellor) for further counselling about their risk level and screening plan. Counselling of women at any risk level is available upon request.

Once notified of their estimated risk level, participants are invited to complete a follow-up questionnaire on the psychological impact of learning their risk level as well as their knowledge, attitudes and behaviors (KAB) towards risk-based screening recommendations. All participants are then invited to complete a 1-year follow-up questionnaire to capture in addition to KAB and psychosocial impact described above, their screening behaviors and short-term outcomes. Linkage to provincial administrative health data will be conducted, allowing for intermediate and long-term follow-up in order to observe the potential impact of risk-based screening on breast screening outcomes (e.g., cancer detection rate, false-positives, benign biopsies, stage at diagnosis). Throughout this pre-implementation study real-world information on human, material and financial resources, and uptake rates is being collected to inform the cost-effectiveness analysis in Activity 4 (see below).

To maximize the long-term impact of this project, a PERSPECTIVE I&I Biobank has been established. This resource provides a platform for the storage of collected data and remaining DNA samples and allows for the possibility to re-contact participants for the collection of additional data or samples for future studies.

#### 2.3.2. Assessing Acceptability and Health Care System Readiness

Implementation of a risk-based screening approach faces considerable organizational challenges that require real-world evidence to address [56,57,58]. To be successful, it also must be accepted and supported by stakeholders, including the beneficiaries (women), providers (health care professionals), and payers (health agencies). To characterize the KAB of Canadian women with respect to risk-based screening, a population-based survey of four Canadian provinces (Alberta, British Columbia, Ontario, Québec) was conducted [59]. This survey assessed attitudes towards risk-based screening including less frequent screening at lower-than-average risk and more frequent screening at high risk, breast cancer risk perception, views on genetic discrimination, knowledge of the potential use of predictive health information by insurers and employers, and perception of the potential impact of breast cancer risk assessment on insurability. A second survey is being developed to examine the knowledge, concerns and needs of clinicians (e.g., medical oncologists, surgeons, radiologists, geneticists, family doctors, and nurses) as they relate to risk prediction, risk communication, and risk-based screening. To make sure that the recruitment and data collection processes presented above are running correctly, we surveyed participants who completed the entry questionnaire and received their saliva kit. In addition, we surveyed physicians and nurse practitioners to obtain their feedback and opinions about receiving risk assessment results for their patients participating in the study. We also developed and planned a series of online forums with health care professionals in order to gather their views on the implementation of a risk-based breast cancer screening approach into the health care system.

A gap analysis will be conducted by reviewing screening program reports, guidelines and policies. Existing screening pathways will be mapped to identify the inter-organizational networks involved, and the resources (human and material) required to operate these programs. Different scenarios for the implementation of a risk-based approach to screening will be developed in consultation with multiple stakeholders and informed by the different components of Activity 3 (e.g., follow-up surveys, health care professionals’ survey etc.). Requirements for risk-based screening will be compared with the existing operational system, ensuring early knowledge transfer and continued preparations for implementation, all in close consultation with stakeholders. These scenarios will also be used to inform simulation models for cost-effectiveness analyses (Activity 4).

#### 2.3.3. Socio-Ethical and Legal Issues

Building on the results from our previous engagement with decision makers [60,61,62], and our extensive research on genetic discrimination [63] and regulatory approval in personalized healthcare [64], potential options to address emergent socio-ethical and legal challenges associated with the implementation of risk-based screening have been identified. These options are being investigated through interdisciplinary legal, ethical and social analyses described below.

As the personalized risk-based approach may involve risk estimation for tens of thousands of women each year, we anticipate that specialized medical health professionals (e.g., geneticists, genetic counsellors) may be reserved for the most complex cases. Decision makers have suggested that nurses could assume increased duties [62], including sampling, requesting laboratory analysis, ordering imaging exams, and providing personalized follow-up recommendations to support this approach. Consideration of this approach is critical both to containing costs and avoiding resource constraints (e.g., genetic counsellors). The legality of this proposal has been analyzed in the current regulatory framework of medical practice [65], and its feasibility and acceptability will be assessed through semi-structured interviews with nurses, other healthcare professionals, and decision-makers.

Once estimated, risk estimates and associated screening action plans must be communicated effectively to women. In-person counselling to understand risk estimates may not always be available (e.g., in remote areas) and may require specialized resources such as phone or video consultations. These resources have been shown to be non-inferior to in-person counseling for knowledge, perceived stress, and satisfaction [66,67], but may provide somewhat lower levels of perceived support and emotional recognition [68]. While video consultations may allow for emotional recognition by providing non-verbal cues, the use of video raises several issues related to acceptability and privacy. The capacity of the current legal framework to regulate this technology [69], as well as women’s satisfaction with virtual care, are being evaluated with selected participants from the PERSPECTIVE I&I cohort.

The use of the extended version of BOADICEA implemented in the CanRisk web-tool [45] raises complex regulatory issues, considering the significance of the medical risk information it provides [51]. The specific requirements needed for the CanRisk tool to comply with the Canadian legal framework for web-tools “software as a Medical Device” are being assessed. The adequacy of this nascent legal framework and its integration with international standards are also evaluated through legal analysis and semi-structured interviews with key stakeholders such as Health Canada’s decision makers and specialists in medical devices and compliance.

One last, but crucial challenge is the need to inform women about the potential risk of discrimination on the basis of genetic and other predictive health data along with existing legal protections [70]. The focus of the Canadian Genetic Non-Discrimination Act S-201 (2017), expressed in the terms used and definition included in the act, is on genetic tests and their results. Hence, the act likely does not apply to other types of predictive health data [71]. Prior to developing resources to inform women of these potential risks, it is crucial to assess their level of understanding of genetic privacy and the potential use of predictive health information by insurers and employers. Concerns about genetic discrimination and, discrimination from predictive results, are assessed in both the population survey described above for other questions [59] and in the follow-up questionnaires (described above).

### 2.4. Economic Analysis to Optimize Personalized Risk-Based Screening Implementation

To determine whether risk-based screening will provide economic value, we are leveraging the BOADICEA algorithm along with the Canadian Partnership Against Cancer (CPAC)-OncoSim Breast Cancer model [72]. While the BOADICEA model estimates individual breast cancer risk, OncoSim Breast Cancer is a microsimulation model that operates at the population level, able to project and evaluate a range of cancer control interventions from screening to treatment. OncoSim is being augmented to simulate a wide range of scenarios where women are offered risk assessment using BOADICEA, and then, based on their risk level, offered more or less intensive breast cancer screening. A significant innovation is the development of the Genetic Mixing Model to provide appropriate population estimates of the joint distribution of the key genetic risk factors–pathogenic variants, family history, and the polygenic risk score [73]. These scenarios will explore policy alternatives regarding the ages at which risk assessments will begin to be offered, the range of data to be used for assessing risk (e.g., whether family history should focus only on first degree relatives or also include second degree), the starting ages and frequencies of breast cancer screening conditional on women’s risk categories, the specific age-dependent thresholds to be used to partition projected risks into different categories, and the modalities for screening (e.g., mammogram, MRI or both).

This expanded OncoSim breast cancer model, known as PopRiskS-BC (Population Risk Stratification for Breast Cancer), simulates breast cancer risk factors and follows a representative population sample of women through screening, diagnosis, treatment, survival and end-of-life care pathways by stage and tumor pathology, competing risks, and quality-adjusted life years (QALYs). The PopRiskS-BC model will also track the costs of all interventions women may encounter along these pathways, including the real-world costs and take-up rates revealed by Activity 3 (described above), in combination with the projected QALYs, to assess the cost-effectiveness of risk-based screening recommendations.

Beyond these simulated data, real-world administrative health data will be available in Ontario, whereas we will use Québec health system costs for comparison. In Ontario the public sector pays for screening, hospitalizations, most physician services, and emergency department services, and for selected prescription medications for all provincial permanent residents (a population of 14.6 million). The provincial government collects individual-level data for all these health service encounters and the providers. The Ontario pre-implementation cohort (Activity 3) will be linked using their unique encrypted health card number to these data in order to determine costs and outcomes related to screening, diagnosis, and treatment [74,75]. The cost of each provincially funded health care resource used by each woman will be calculated, as well as the health care resources used by the risk stratification procedures themselves.

Finally, the PopRiskS-BC model will be used with the administrative data to evaluate the prospective cost-effectiveness, budget, and other impacts of different screening scenarios (e.g., variable start age, frequency, modality based on risk). This analysis will be conducted in accordance with the recent update to the Canadian Agency for Drugs and Technologies in Health (CADTH) guidelines (29 March 2017) for cost-effectiveness evaluations [2].

## 3. Discussion

Early randomized clinical trials [76] showed that screening with mammography reduces breast cancer mortality [8,77], and led to the widespread implementation of population-based screening programs around the world. Both the Canadian Task Force on Preventive Health Care [6] and the US Preventive Services Task Force [78] recommend breast screening every two to three years for women age 50 to 74 years. The benefits of screening mammography however, come with potential harms, including anxiety and unnecessary work-up associated with a false-positive result, or a false-negative result leading to false reassurance and delayed diagnosis, as well as overdiagnosis [79,80,81]. Furthermore, because breast cancer risk is variable within the population, as is the sensitivity of mammography (e.g., lower sensitivity in women with dense breasts [82,83]), different women ideally require different recommendations regarding the most appropriate screening plan for their personal situation. This could include recommendations for screening frequency, age of screening initiation and cessation, and the use of additional screening modalities (e.g., MRI, ultrasound), and potentially consideration of preventive treatments.

The PERSPECTIVE I&I project has assembled a multidisciplinary team of researchers, clinicians and decision makers to build on best practices, generate evidence to address the challenges associated with implementation of a risk-based approach within existing screening programs, as well as assessing the impact on patient outcomes and experience. This project takes a comprehensive approach to improving the accuracy of breast cancer risk assessment. In the cancer genetic clinic, this will optimize the genetic counselling of women at high risk with respect to screening and risk reduction strategies. At the population-level, this will generate real-world evidence on how to shift from a primarily age-based screening approach to one based on estimated personalized risk. This includes assessment of the feasibility, organizational readiness, effectiveness, efficiency, resources, costs and cost-effectiveness of implementing a risk-based breast cancer screening approach within the Canadian healthcare system.

This interdisciplinary project has several strengths. The first is our approach to the identification, validation, and integration of both rare and common genetic variation underlying breast cancer risk, to develop a clinical grade genetic test. This work will inform risk assessment in women suspected to be at high risk who are seen in cancer genetic clinics, but also extend this approach to the general population. To this end, conducting the pre-implementation study using the infrastructure of existing screening programs as opposed to developing a new system to support a risk-based approach is a major strength. Furthermore, by incorporating the preferences of women and how knowledge of risk affects behavior, as well as considering the legal implications of changing clinical roles, risk communication and privacy, this work will inform our understanding of the experience of both the healthcare system and women undergoing risk assessment. Moreover, leveraging available administrative health data resources allows for long-term follow-up to assess the impact of a risk-based approach on outcomes, while considering issues related to cost-effectiveness.

Still, this study has some limitations. First, while it is a strength that we are conducting the pre-implementation study (Activity 3) in two Canadian provinces representing a little over 60% of the population of Canada, allowing us to examine the impact of different recruitment methods and healthcare delivery systems, this does not capture potential variability present within other provinces and territories. This is partially being mitigated through the creation of a Canadian Translational Advisory Committee with national representation that meets biannually to facilitate translation of knowledge produced by PERSPECTIVE I&I project and uptake of project findings. We are also only including women who have had a prior mammogram, to allow for the inclusion of mammographic density in the risk prediction calculation. Practically, many younger women (prior to age 50 years) may have their risk estimated prior to having a screening mammogram, in particular those referred to genetic clinics for risk assessment (and referral for to the High Risk Ontario Breast Screening Program) as they may be at high risk. To address this issue for implementation, risks will be estimated excluding different risk factors (including mammographic density) to determine the potential impact on risk estimation and any associated screening recommendations.

The European Collaborative on Personalized Early Detection and Prevention of Breast Cancer (ENVISION) recently published a consensus statement [28] highlighting the need for implementation studies that address feasibility and acceptability, including health system readiness, engaging multiple stakeholders, and considering social/ethical/legal issues related to risk prediction and risk communication. To address this need, in addition to our project in Canada (PERSPECTIVE I&I), efforts are underway in the United States (WISDOM [84,85]), Europe (e.g., My Personal Breast Screening [MyPebs] [86]), and the United Kingdom (Predicting the Risk of Cancer at Screening [PROCAS] [87]) to use risk prediction models to inform recommendations for screening and prevention [28]. The collective efforts of these projects will help to generate evidence for a risk-based approach with the goal of improving the benefit-harm ratio of population-based breast cancer screening programs across all levels of risk.

Ideally, risk-based screening will balance the benefits and harms of screening by targeting higher risk women to receive earlier and more frequent breast cancer screening, in turn ensuring they preferentially receive timely diagnosis and treatment. Simultaneously, women at lower risk may avoid unnecessary procedures and treatments that could lead to complications and psychological stress. The microsimulation results will inform whether and how to screen low-risk women. This project will generate the necessary evidence to support such a more personalized screening program design. Furthermore, accurate risk assessment may provide an opportunity for the reallocation of resources toward those who will benefit the most, increasing efficiency of an already resource constrained healthcare system [88]. Ultimately, it is hoped that personalized risk assessment and risk communication will offer more women the opportunity to make informed choices with their health care provider about effective breast cancer screening and prevention approaches and improve their health empowerment.

## Figures and Tables

**Table 1:** Overview of screening action plan, risk communication & follow-up procedures by estimated risk level.

Risk Level	Risk Communication ^a^	Screening Action Plan	Follow-Up
Average 10-year absolute risk equivalent to lifetime risk of <15%	Risk Letter: includes risk level and range	40–49 years: No regular screening with a mammogram	Phone call with study genetic counsellor or nurse available upon request
	*Average risk: “In this risk level, up to <number> out of 1000 women your age may get breast cancer over the next 10 years”.*	50–69 years: Screened every 2 years with a mammogram	Follow-up with questionnaire at time of risk communication and 1 year later.
Higher than Average 10-year absolute risk equivalent to lifetime risk of 15 to <25%	Risk Letter: includes risk level and range	Ontario 40–49 years: Talk to primary care provider about screening with a mammogram every year.	Phone call with study genetic counsellor or nurse available upon request
	*Higher than Average risk: “In this risk level, about <number> to <number> out of 1000 women your age may get breast cancer over the next 10 years”.*	50–69 years: Screened every year with a mammogram	Follow-up with questionnaire at time of risk communication and 1 year later.
		Québec Screened every 1–2 years with a mammogram, ultrasound considered if breast density is >75%	
High 10-year absolute risk equivalent to lifetime risk of ≥25%	Risk Letter: includes risk level and range	40–69 years: Screened every year with a mammogram and magnetic resonance imaging (MRI)	Phone call with study genetic counsellor or nurse
	*High risk: “In this risk level, <number> or more out of 1000 women your age may get breast cancer over the next 10 years”.*		Follow-up with questionnaire at time of risk communication and 1 year later.

^a^ Women are encouraged to discuss their risk level and screening action plan with their primary care provider. In Quebec, the risk letter is also sent to the family physician. Additional materials supporting risk communication for both women and their primary care provider include: A booklet/Information sheet on Understanding your Assessment, the study Website also includes information to support understanding your risk assessment as well as many other resources (e.g., frequently asked questions).

## Data Availability

Data sharing not applicable.

## References

[1] Canadian Cancer Statistics Advisory Committee Canadian Cancer Statistics 2019. http://cancer.ca/Canadian-Cancer-Statistics-2019-EN.

[2] Guidelines for the Economic Evaluation of Health Technologies: Canada. https://www.cadth.ca/about-cadth/how-we-do-it/methods-and-guidelines/guidelines-for-the-economic-evaluation-of-health-technologies-canada.

[3] De Oliveira C., Pataky R., Bremner K.E., Rangrej J., Chan K.K., Cheung W.Y., Hoch J.S., Peacock S., Krahn M.D. (2016). Phase-specific and lifetime costs of cancer care in Ontario, Canada. BMC Cancer.

[4] Patra J., Popova S., Rehm J., Bondy S., Flint R., Giesbrecht N. Economic cost of chronic disease in Canada. https://www.ocdpa.ca/sites/default/files/publications/OCDPA_EconomicCosts.pdf.

[5] Will B., Berthelot J.-M., Le Petit C., Tomiak E., Verma S., Evans W. (2000). Estimates of the lifetime costs of breast cancer treatment in Canada. Eur. J. Cancer.

[6] Klarenbach S., Sims-Jones N., Lewin G., Singh H., Thériault G., Tonelli M., Doull M., Courage S., Garcia A.J., Thombs B.D. (2018). Recommendations on screening for breast cancer in women aged 40–74 years who are not at increased risk for breast cancer. Can. Med. Assoc. J..

[7] Korenstein D. (2018). Wise guidance and its challenges: The new Canadian recommendations on breast cancer screening. Can. Med. Assoc. J..

[8] Lauby-Secretan B., Scoccianti C., Loomis D., Benbrahim-Tallaa L., Bouvard V., Bianchini F., Straif K. (2015). Breast-Cancer Screening—Viewpoint of the IARC Working Group. N. Engl. J. Med..

[9] Sutton S., Saidi G., Bickler G., Hunter J. (1995). Does routine screening for breast cancer raise anxiety? Results from a three wave prospective study in England. J. Epidemiol. Community Health.

[10] Puliti D., Miccinesi G., Paci E. (2011). Overdiagnosis in breast cancer: Design and methods of estimation in observational studies. Prev. Med..

[11] Pharoah P.D., Antoniou A., Bobrow M., Zimmern R.L., Easton D.F., Ponder B.A. (2002). Polygenic susceptibility to breast cancer and implications for prevention. Nat. Genet..

[12] Pharoah P.D., Antoniou A.C., Easton D.F., Ponder B.A. (2008). Polygenes, risk prediction, and targeted prevention of breast cancer. N. Engl. J. Med..

[13] Chiarelli A.M., Prummel M.V., Muradali D., Majpruz V., Horgan M., Carroll J.C., Eisen A., Meschino W.S., Shumak R.S., Warner E. (2014). Effectiveness of screening with annual magnetic resonance imaging and mammography: Results of the initial screen from the ontario high risk breast screening program. J. Clin. Oncol..

[14] Canadian Partnership Against Cancer (2017). Breast Cancer Screening in Canada: Monitoring and Evaluation of Quality Indicator—Results Report, January 2011 to December 2012.

[15] Chiarelli A.M., Blackmore K.M., Muradali D., Done S.J., Majpruz V., Weerasinghe A., Mirea L., Eisen A., Rabeneck L., Warner E. (2019). Performance Measures of Magnetic Resonance Imaging Plus Mammography in the High Risk Ontario Breast Screening Program. J. Natl. Cancer Inst..

[16] National Comprehensive Cancer Network (NCCN) NCCN Guidelines Version 1.2019 Breast Cancer Screening and Diagnosis. www.nccn.org.

[17] National Institute of Health and Care Excellence (2013). Familial Breast Cancer: Classification and Care of People at Risk of Familial Breast Cancer and Management of Breast Cancer and Related Risks in People with a Family History of Breast Cancer.

[18] Saslow D., Boetes C., Burke W., Harms S., Leach M.O., Lehman C.D., Morris E., Pisano E., Schnall M., Sener S. (2007). American Cancer Society Guidelines for Breast Screening with MRI as an Adjunct to Mammography. CA Cancer J. Clin..

[19] Easton D.F., Pharoah P.D.P., Antoniou A.C., Tischkowitz M., Tavtigian S.V., Nathanson K.L., Devilee P., Meindl A., Couch F.J., Southey M. (2015). Gene-Panel Sequencing and the Prediction of Breast-Cancer Risk. N. Engl. J. Med..

[20] Dorling L., Carvalho S., Allen J., González-Neira A., Luccarini C., Wahlström C., Pooley K.A., Parsons M.T., Fortuno C., Wang Q. (2021). Breast Cancer Risk Gene—Association Analysis in More than 113,000 Women. N. Engl. J. Med..

[21] Hu C., Hart S.N., Gnanaolivu R., Huang H., Lee K.Y., Na J., Gao C., Lilyquist J., Yadav S., Boddicker N.J. (2021). A Population-Based Study of Genes Previously Implicated in Breast Cancer. N. Engl. J. Med..

[22] Michailidou K., Lindström S., Dennis J., Beesley J., Hui S., Kar S., Lemaçon A., Soucy P., Glubb D., Rostamianfar A. (2017). Association analysis identifies 65 new breast cancer risk loci. Nature.

[23] Mavaddat N., Michailidou K., Dennis J., Lush M., Fachal L., Lee A., Tyrer J.P., Chen T.-H., Wang Q., Bolla M.K. (2019). Polygenic risk scores for prediction of breast cancer and breast cancer subtypes. Am. J. Hum. Genet..

[24] Choudhury P., Brook M.N., Hurson A.N., Lee A., Mulder C.V., Coulson P., Schoemaker M.J., Jones M.E., Swerdlow A.J., Chatterjee N. (2021). Comparative validation of the BOADICEA and Tyrer-Cuzick breast cancer risk models incorporating classical risk factors and polygenic risk in a population-based prospective cohort of women of European ancestry. Breast Cancer Res..

[25] Lakeman I.M.M., Rodríguez-Girondo M., Lee A., Ruiter R., Stricker B.H., Wijnant S.R.A., Kavousi M., Antoniou A.C., Schmidt M.K., Uitterlinden A.G. (2020). Validation of the BOADICEA model and a 313-variant polygenic risk score for breast cancer risk prediction in a Dutch prospective cohort. Genet. Med..

[26] Lee A., Mavaddat N., Wilcox A.N., Cunningham A.P., Carver T., Hartley S., Babb de Villiers C., Izquierdo A., Simard J., Schmidt M.K. (2019). BOADICEA: A comprehensive breast cancer risk prediction model incorporating genetic and nongenetic risk factors. Genet. Med..

[27] Terry M.B., Liao Y., Whittemore A.S., Leoce N., Buchsbaum R., Zeinomar N., Dite G.S., Chung W.K., Knight J.A., Southey M.C. (2019). 10-year performance of four models of breast cancer risk: A validation study. Lancet Oncol..

[28] Pashayan N., Antoniou A.C., Ivanus U., Esserman L.J., Easton D.F., French D., Sroczynski G., Hall P., Cuzick J., Evans D.G. (2020). Personalized early detection and prevention of breast cancer: ENVISION consensus statement. Nat. Rev. Clin. Oncol..

[29] Nepomuceno T.C., Carvalho M.A., Rodrigue A., Simard J., Masson J.Y., Monteiro A.N.A. (2020). PALB2 Variants: Protein Domains and Cancer Susceptibility. Trends Cancer.

[30] Rodrigue A., Margaillan G., Torres Gomes T., Coulombe Y., Montalban G., da Costa E.S.C.S., Milano L., Ducy M., De-Gregoriis G., Dellaire G. (2019). A global functional analysis of missense mutations reveals two major hotspots in the PALB2 tumor suppressor. Nucleic Acids Res..

[31] Wiltshire T., Ducy M., Foo T.K., Hu C., Lee K.Y., Belur Nagaraj A., Rodrigue A., Gomes T.T., Simard J., Monteiro A.N.A. (2020). Functional characterization of 84 PALB2 variants of uncertain significance. Genet. Med..

[32] Boonen R.A.C.M., Rodrigue A., Stoepker C., Wiegant W.W., Vroling B., Sharma M., Rother M.B., Celosse N., Vreeswijk M.P.G., Couch F. (2019). Functional analysis of genetic variants in the high-risk breast cancer susceptibility gene PALB2. Nat. Commun..

[33] Findlay G.M., Boyle E.A., Hause R.J., Klein J.C., Shendure J. (2014). Saturation editing of genomic regions by multiplex homology-directed repair. Nature.

[34] Fachal L., Aschard H., Beesley J., Barnes D.R., Allen J., Kar S., Pooley K.A., Dennis J., Michailidou K., Turman C. (2020). Fine-mapping of 150 breast cancer risk regions identifies 191 likely target genes. Nat. Genet..

[35] Milne R.L., Kuchenbaecker K.B., Michailidou K., Beesley J., Kar S., Lindström S., Hui S., Lemaçon A., Soucy P., Dennis J. (2017). Identification of ten variants associated with risk of estrogen-receptor-negative breast cancer. Nat. Genet..

[36] Zhang H., Ahearn T.U., Lecarpentier J., Barnes D., Beesley J., Qi G., Jiang X., O’Mara T.A., Zhao N., Bolla M.K. (2020). Genome-wide association study identifies 32 novel breast cancer susceptibility loci from overall and subtype-specific analyses. Nat. Genet..

[37] Barnes D.R., Rookus M.A., McGuffog L., Leslie G., Mooij T.M., Dennis J., Mavaddat N., Adlard J., Ahmed M., Aittomäki K. (2020). Polygenic risk scores and breast and epithelial ovarian cancer risks for carriers of BRCA1 and BRCA2 pathogenic variants. Genet. Med..

[38] Maas P., Barrdahl M., Joshi A.D., Auer P.L., Gaudet M.M., Milne R.L., Schumacher F.R., Anderson W.F., Check D., Chattopadhyay S. (2016). Breast Cancer Risk From Modifiable and Nonmodifiable Risk Factors Among White Women in the United States. JAMA Oncol..

[39] Kapoor P.M., Mavaddat N., Choudhury P.P., Wilcox A.N., Lindström S., Behrens S., Michailidou K., Dennis J., Bolla M.K., Wang Q. (2020). Combined Associations of a Polygenic Risk Score and Classical Risk Factors With Breast Cancer Risk. J. Natl. Cancer Inst..

[40] Antoniou A.C., Cunningham A.P., Peto J., Evans D.G., Lalloo F., Narod S.A., Risch H.A., Eyfjord J.E., Hopper J.L., Southey M.C. (2008). The BOADICEA model of genetic susceptibility to breast and ovarian cancers: Updates and extensions. Br. J. Cancer.

[41] Lee A.J., Cunningham A.P., Tischkowitz M., Simard J., Pharoah P.D., Easton D.F., Antoniou A.C. (2016). Incorporating truncating variants in PALB2, CHEK2, and ATM into the BOADICEA breast cancer risk model. Genet. Med..

[42] MacInnis R., Bickerstaffe A., Apicella C., Dite G.S., Dowty J.G., Aujard K., Phillips K., Weideman P., Lee A., Terry M.B. (2013). Prospective validation of the breast cancer risk prediction model BOADICEA and a batch-mode version BOADICEACentre. Br. J. Cancer.

[43] Ho W.-K., Tan M.-M., Mavaddat N., Tai M.-C., Mariapun S., Li J., Ho P.-J., Dennis J., Tyrer J.P., Bolla M.K. (2020). European polygenic risk score for prediction of breast cancer shows similar performance in Asian women. Nat. Commun..

[44] Fortier I., Dragieva N., Saliba M., Craig C., Robson P.J. (2019). Harmonization of the Health and Risk Factor Questionnaire Data of the Canadian Partnership for Tomorrow Project: A descriptive analysis. CMAJ Open.

[45] Carver T., Hartley S., Lee A., Cunningham A.P., Archer S., Babb de Villiers C., Roberts J., Ruston R., Walter F.M., Tischkowitz M. (2020). CanRisk Tool—A Web Interface for the Prediction of Breast and Ovarian Cancer Risk and the Likelihood of Carrying Genetic Pathogenic Variants. Cancer Epidemiol. Biomark. Prev..

[46] Archer S., Babb de Villiers C., Scheibl F., Carver T., Hartley S., Lee A., Cunningham A.P., Easton D.F., McIntosh J.G., Emery J. (2020). Evaluating clinician acceptability of the prototype CanRisk tool for predicting risk of breast and ovarian cancer: A multi-methods study. PLoS ONE.

[47] Regulation (EU) 2017/745 of the European Parliament and of the Council on medical devices. https://eur-lex.europa.eu/legal-content/EN/TXT/?uri=CELEX%3A32017R0745.

[48] Regulation (EU) 2017/746 of the European Parliament and of the Council on in vitro Diagnostic Medical Devices. https://eur-lex.europa.eu/legal-content/EN/TXT/?uri=CELEX:32017R0746.

[49] Medical Devices Regulations, SOR/98-282. https://laws-lois.justice.gc.ca/eng/regulations/sor-98-282/.

[50] European Commission Guidelines on the Qualification and Classification of Stand Alone Software Used in Healthcare within the Regulatory Framework of Medical Devices. https://ec.europa.eu/docsroom/documents/17921.

[51] Health Canada Guidance Document Software as Medical Device (SaMD). Definition and Classification. (Canada: Health Canada, 2019). https://www.canada.ca/en/health-canada/services/drugs-health-products/medical-devices/application-information/guidance-documents/software-medical-device-guidance-document.html.

[52] Thorogood A., Touré S.B., Ordish J., Hall A., Knoppers B. (2018). Genetic database software as medical devices. Hum. Mutat..

[53] Chiarelli A.M., Blackmore K.M., Mirea L., Done S.J., Majpruz V., Weerasinghe A., Rabeneck L., Muradali D. (2020). Annual vs Biennial Screening: Diagnostic Accuracy Among Concurrent Cohorts Within the Ontario Breast Screening Program. J. Natl. Cancer Inst..

[54] Perron L., Chang S.L., Daigle J.M., Vandal N., Theberge I., Diorio C., Lemieux J., Pelletier E., Brisson J. (2019). Breast cancer subtype and screening sensitivity in the Quebec Mammography Screening Program. J. Med. Screen..

[55] Green L., Kreuter M. (2005). Health Program Planning: An Educational and Ecological Approach.

[56] Dent T., Jbilou J., Rafi I., Segnan N., Törnberg S., Chowdhury S., Hall A., Lyratzopoulos G., Eeles R., Eccles D. (2013). Stratified cancer screening: The practicalities of implementation. Public Health Genom..

[57] Haas J.S. (2017). The complexity of achieving the promise of precision breast cancer screening. J. Natl. Cancer Inst..

[58] Marcus P.M., Pashayan N., Church T.R., Doria-Rose V.P., Gould M.K., Hubbard R.A., Marrone M., Miglioretti D.L., Pharoah P.D., Pinsky P.F. (2016). Population-based precision cancer screening: A symposium on evidence, epidemiology, and next steps. Cancer Epidemiol. Biomark. Prev..

[59] Mbuya Bienge C., Pashayan N., Brooks J.D., Dorval M., Chiquette J., Eloy L., Turgeon A., Lambert-Côté L., Paquette J.S., Lévesque E. (2021). Women’s Views on Multifactorial Breast Cancer Risk Assessment and Risk-Stratified Screening: A Population-Based Survey from Four Provinces in Canada. J. Pers. Med..

[60] Hagan J., Lévesque E., Knoppers B.M. (2016). Influence of organizational factors on implementation of a personalized approach to breast cancer screening. Sante Publique.

[61] Lévesque E., Hagan J., Knoppers B.M., Simard J. (2019). Organizational challenges to equity in the delivery of services within a new personalized risk-based approach to breast cancer screening. New Genet. Soc..

[62] Esquivel-Sada D., Lévesque E., Hagan J., Knoppers B., Simard J. (2019). Envisioning Implementation of a Personalized Approach in Breast Cancer Screening Programs: Stakeholder Perspectives. Healthc. Policy.

[63] Dalpé G., Ngueng Feze I., Salman S., Joly Y., Hagan J., Lévesque E., Dorval V., Blouin-Bougie J., Amara N., Dorval M. (2017). Breast cancer risk estimation and personal insurance: A qualitative study presenting perspectives from canadian patients and decision makers. Front. Genet..

[64] Joly Y., Koutrikas G., Tasse A.-M., Issa A. (2011). Regulatory approval for new pharmacogenomic tests: A comparative overview. Food Drug Law J..

[65] Lévesque E., Knoppers B.M. (2021). Faire Jouer un Rôle Élargi aux Infirmières Dans une Approche Individualisée de Dépistage du Cancer du Sein: Analyse des Options Juridiques.

[66] Schwartz M.D., Valdimarsdottir H.B., Peshkin B.N., Mandelblatt J., Nusbaum R., Huang A.-T., Chang Y., Graves K., Isaacs C., Wood M. (2014). Randomized noninferiority trial of telephone versus in-person genetic counseling for hereditary breast and ovarian cancer. J. Clin. Oncol..

[67] Kinney A.Y., Steffen L.E., Brumbach B.H., Kohlmann W., Du R., Lee J.H., Gammon A., Butler K., Buys S.S., Stroup A.M. (2016). Randomized Noninferiority Trial of Telephone Delivery of BRCA1/2 Genetic Counseling Compared With In-Person Counseling: 1-Year Follow-Up. J. Clin. Oncol..

[68] Peshkin B.N., Kelly S., Nusbaum R.H., Similuk M., DeMarco T.A., Hooker G.W., Valdimarsdottir H.B., Forman A.D., Joines J.R., Davis C. (2016). Patient perceptions of telephone vs. in-person BRCA1/BRCA2 genetic counseling. J. Genet. Couns..

[69] Lévesque E., Knoppers B.M. (2021). La Télésanté́ au Québec: Quel Encadrement Pour la Consultation Vidéo? Revue de Droit de l’Université́ de Sherbrooke.

[70] Joly Y., Dalpé G., Dupras C., Bévière-Boyer B., de Paor A., Dove E.S., Granados Moreno P., Ho C.W.L., Ho C.H., Cathaoir K.Ó. (2020). Establishing the International Genetic Discrimination Observatory. Nat. Genet..

[71] Joly Y., Dalpé G., Pinkesz M. (2019). Is Genetic Discrimination Back on the Radar? A Commentary on the Recent Court of Appeal Reference Decision on the Genetic Non-Discrimination Act (GNDA). Can. J. Bioeth./Revue Can. Bioéth..

[72] Gauvreau C.L., Fitzgerald N.R., Memon S., Flanagan W.M., Nadeau C., Asakawa K., Garner R., Miller A.B., Evans W.K., Popadiuk C.M. (2017). The OncoSim model: Development and use for better decision-making in Canadian cancer control. Curr. Oncol..

[73] Wolfson M., Gribble S., Pashayan N., Easton D.F., Antoniou A.C., Lee A., van Katwyk S., Simard J. Potential of Polygenic Risk Scores for Improving Population Estimates of Women’s Breast Cancer Genetic Risks. Genet. Med..

[74] Mittmann N., Stout N.K., Lee P., Tosteson A.N., Trentham-Dietz A., Alagoz O., Yaffe M.J. (2015). Total cost-effectiveness of mammography screening strategies. Health Rep..

[75] Mittmann N., Stout N.K., Tosteson A.N., Trentham-Dietz A., Alagoz O., Yaffe M.J. (2018). Cost-effectiveness of mammography from a publicly funded health care system perspective. CMAJ Open.

[76] Shapiro S. (1977). Evidence on screening for breast cancer from a randomized trial. Cancer.

[77] Coldman A., Phillips N., Wilson C., Decker K., Chiarelli A.M., Brisson J., Zhang B., Payne J., Doyle G., Ahmad R. (2014). Pan-Canadian study of mammography screening and mortality from breast cancer. J. Natl. Cancer Inst..

[78] Nelson H.D., Fu R., Cantor A., Pappas M., Daeges M., Humphrey L. (2016). Effectiveness of Breast Cancer Screening: Systematic Review and Meta-analysis to Update the 2009 U.S. Preventive Services Task Force Recommendation. Ann. Intern. Med..

[79] Brennan M., Houssami N. (2016). Discussing the benefits and harms of screening mammography. Maturitas.

[80] Gøtzsche P.C., Jørgensen K.J. (2013). Screening for breast cancer with mammography. Cochrane Database Syst. Rev..

[81] Myers E.R., Moorman P., Gierisch J.M., Havrilesky L.J., Grimm L.J., Ghate S., Davidson B., Mongtomery R.C., Crowley M.J., McCrory D.C. (2015). Benefits and Harms of Breast Cancer Screening: A Systematic Review. JAMA.

[82] Ma L., Fishell E., Wright B., Hanna W., Allan S., Boyd N.F. (1992). Case-Control Study of Factors Associated With Failure to Detect Breast Cancer by Mammography. J. Natl. Cancer Inst..

[83] Mandelson M.T., Oestreicher N., Porter P.L., White D., Finder C.A., Taplin S.H., White E. (2000). Breast Density as a Predictor of Mammographic Detection: Comparison of Interval- and Screen-Detected Cancers. J. Natl. Cancer Inst..

[84] Esserman L.J. (2017). The WISDOM Study: Breaking the deadlock in the breast cancer screening debate. NPJ Breast Cancer.

[85] Shieh Y., Eklund M., Madlensky L., Sawyer S.D., Thompson C.K., Stover Fiscalini A., Ziv E., Van’t Veer L.J., Esserman L.J., Tice J.A. (2017). Breast Cancer Screening in the Precision Medicine Era: Risk-Based Screening in a Population-Based Trial. J. Natl. Cancer Inst..

[86] The Project—MyPeBS. https://mypebs.eu/the-project/.

[87] Evans D.G., Astley S., Stavrinos P., Harkness E., Donnelly L.S., Dawe S., Jacob I., Harvie M., Cuzick J., Brentnall A. (2016). Programme Grants for Applied Research. Improvement in Risk prediction, Early Detection and Prevention of Breast Cancer in the NHS Breast Screening Programme and Family History Clinics: A Dual Cohort Study.

[88] Pashayan N., Morris S., Gilbert F.J., Pharoah P.D.P. (2018). Cost-effectiveness and Benefit-to-Harm Ratio of Risk-Stratified Screening for Breast Cancer: A Life-Table Model. JAMA Oncol..

